# Thoughts about *SLC16A2*, *TSIX* and *XIST* gene like sites in the human genome and a potential role in cellular chromosome counting

**DOI:** 10.1186/s13039-016-0271-7

**Published:** 2016-08-08

**Authors:** Martina Rinčić, Ivan Y. Iourov, Thomas Liehr

**Affiliations:** 1 Croatian Institute for Brain Research, School of Medicine University of Zagreb, Salata 12, 10000 Zagreb, Croatia; 2 Mental Health Research Center, Moscow, 117152 Russia; 3 Ministry of Health of Russian Federation, Separated Structural Unit “Clinical Research Institute of Pediatrics” at Pirogov Russian National Research Medical University, Moscow, 125412 Russia; 4 Moscow State University of Psychology and Education, Moscow, 127051 Russia; 5 Institute of Human Genetics, Jena University Hospital, Friedrich Schiller University, Kollegiengasse 10, D-07743 Jena, Germany

**Keywords:** Chromosome counting, X-chromosome inactivation, Chromosome kissing, Chromosome territories, Sequence similarity, *SLC16A2*, *TSIX*, *XIST*

## Abstract

**Background:**

Chromosome counting is a process in which cells determine somehow their intrinsic chromosome number(s). The best-studied cellular mechanism that involves chromosome counting is ‘chromosome-kissing’ and X-chromosome inactivation (XCI) mechanism. It is necessary for the well-known dosage compensation between the genders in mammals to balance the number of active X-chromosomes (Xa) with regard to diploid set of autosomes. At the onset of XCI, two X-chromosomes are coming in close proximity and pair physically by a specific segment denominated X-pairing region (Xpr) that involves the *SLC16A2* gene.

**Results:**

An Ensembl BLAST search for human and mouse *SLC16A2*/*Slc16a2* homologues revealed, that highly similar sequences can be found at almost each chromosome in the corresponding genomes. Additionally, a BLAST search for *SLC16A2*/*TSIX*/*XIST* (genes responsible for XCI) reveled that “*SLC16A2*/*TSIX*/*XIST* like sequences” cover equally all chromosomes, too. With respect to this we provide following hypotheses.

**Hypotheses:**

If a single genomic region containing the *SLC16A2* gene on X-chromosome is responsible for maintaining “balanced” active copy numbers, it is possible that similar sequences or gene/s have the same function on other chromosomes (autosomes). *SLC16A2* like sequences on autosomes could encompass evolutionary older, but functionally active key regions for chromosome counting in early embryogenesis. Also *SLC16A2* like sequence on autosomes could be involved in inappropriate chromosomes pairing and, thereby be involved in aneuploidy formation during embryogenesis and cancer development. Also, “*SLC16A2/TSIX/XIST* gene like sequence combinations” covering the whole genome, could be important for the determination of X:autosome ratio in cells and chromosome counting.

**Conclusions:**

*SLC16A2* and/or *SLC16A2/TSIX/XIST* like sequence dispersed across autosomes and X-chromosome(s) could serve as bases for a counting mechanism to determine X:autosome ratio and could potentially be a mechanism by which a cell also counts its autosomes. It could also be that such specific genomic regions have the same function for each specific autosome. As errors during the obviously existing process of chromosome counting are one if not the major origin of germline/somatic aneuploidy the here presented hypotheses should further elaborated and experimentally tested.

**Electronic supplementary material:**

The online version of this article (doi:10.1186/s13039-016-0271-7) contains supplementary material, which is available to authorized users.

## Background

**Fig. 1:**
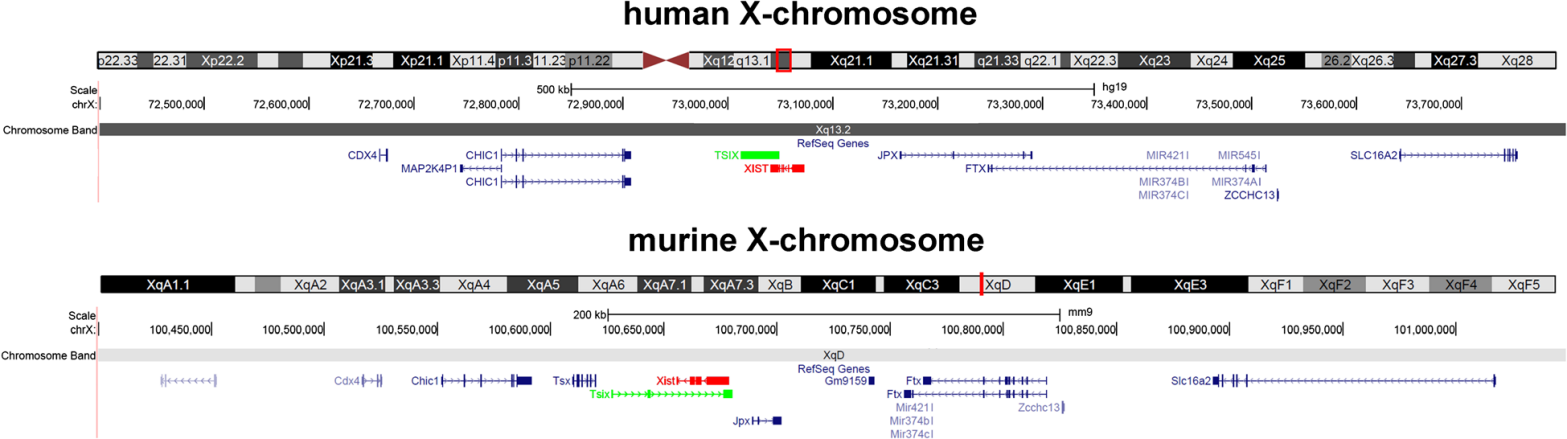
The two mayor players of X-chromosome inactivation. The localization of X-inactivation center (XIC) on human and murine X- chromosome ideogram is highlighted in red on the depiction of the corresponding entire X-chromosomes. The highlighted XIC-containing region of human (Xq13.2) and murine X-chromosome (XqD) is enlarged and depicted below the corresponding ideograms. *XIST*/*Xist* region is again highlighted for this magnification in red and shown together with other transcripts of this region. *XIST*/*Xist* encodes a nontranslated nuclear RNA which spreads along the X-chromosome and initiates silencing. *TSIX*/*Tsix* (highlighted in green) creates an antisense RNA spanning all of *XIST*/*Xist* region enabling prevention of *XIST*/*Xist* RNA spreading on future Xa (active X-chromosome)

**Fig. 2:**
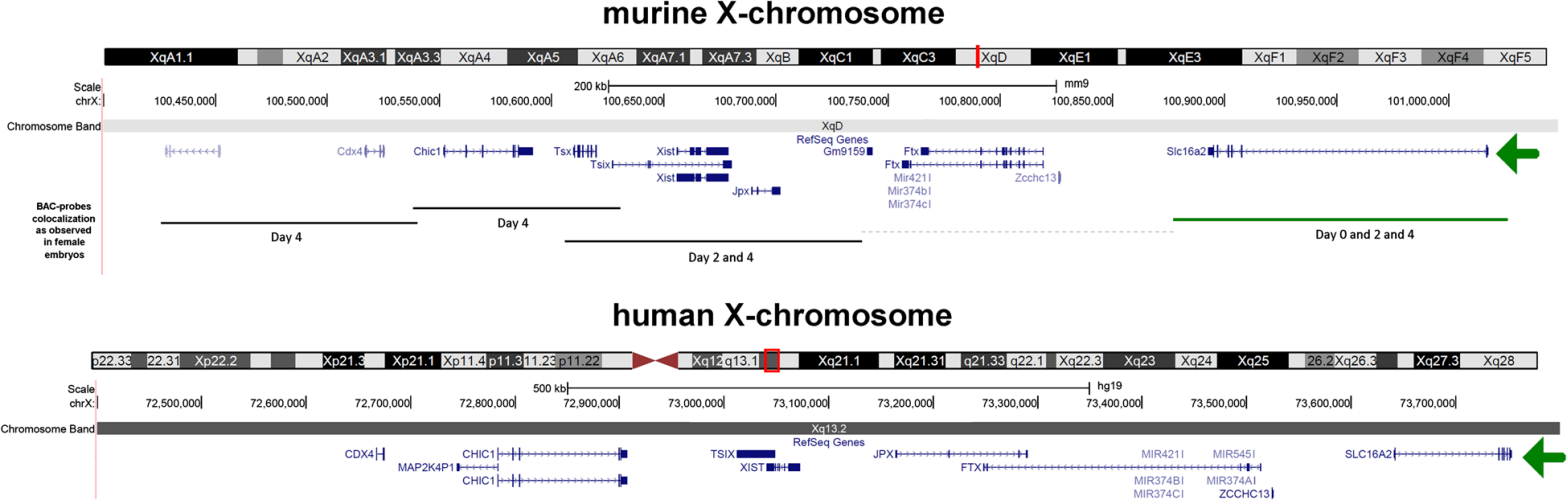
The X-Pairing Region (*Xpr*). Here are summarized data from Augui et al. [10], who used bacterial artificial chromosome (BAC) probes to study interallelic X-chromosome distances at different stages of murine female embryogenesis. The X-inactivation center (XIC), or at least a part of it, is paring in murine female embryo from day 0 on (in undifferentiated ES cells) – green line. This genomic segment contains most of the *Slc16a2* gene and was denominated X-pairing region (*Xpr*). After this segment paired, other parts of this region marked with black lines were pairing. XIC was paired within a critical time window on day 2 in which *Xist* becomes monoallelically up-regulated, taking place after *Xpr* aligned. No corresponding study is available for humans; however, homologous human region is depicted for comparison. *SLC16A2* gene is highlighted for human and mouse by a green arrow

X-chromosome inactivation (XCI) is a process by which mammals, or better their cells, balance the number of active X-chromosomes (Xa) with regard to a diploid set of autosomes. Dosage compensation between genders in mammals is achieved by keeping only one Xa per diploid set of autosomes. Therefore the majority of genes on one of the two X-chromosomes in female mammals is silenced and denoted as inactive X-chromosome (Xi) or Barr body [1]. What is known about molecular mechanism of XCI was raised from the most popular mammalian genetic research model *Mus musculus* (“laboratory mice”) [2]. From the discovery of a single genomic locus that is the starting point (“initial spot”) of XCI (later on called X inactivation center – XIC), underlying mechanisms were extensively studied [3, 4]. XIC is a small region on the X-chromosome that contains elements being crucial for XCI process (Fig. 1). This process leads in the end to an epigenetic modification of one of the X-chromosomes, starting from XIC; this process was divided into four stages: initiation, speeding, maintenance and reactivation [5]. The initiation stage of XCI includes as two most important steps counting and choosing. ‘Counting’ is in a way a process by which cells measure the X:autosome ratio and ‘choosing’ is the process that identifies which X-chromosome is to be inactivated. The idea that counting mechanism exists was provided during the early years of cytogenetics based on simple observations on cells with abnormal sex chromosome numbers (gonosomal aneuploidy). In females, diploid cells with more than two X-chromosomes inactivate all but one of them, as in contrast cells with 45,X- or 46,XY-karyotypes do not undergo XCI [6]. Although molecular mechanisms were not known at that time, this was already a proof by evidence that cells can count and exactly determine the number of their X-chromosomes. An involvement of autosomes in X-chromosome could be suggested after discovery of two Xa and two Xi in tetraploid cells [7]. Although extensively studied, molecular mechanism/s that underlie counting and choosing are still poorly understood. Most of the efforts for finding a sequence being responsible for sensing and counting were focused on a “small” region that encompasses the noncoding RNAs in XIC (Fig. 1). This search has pointed out specific segments that were implied as counting factor, namely RNA anti-sense to *Xist* [8]. At the onset of random inactivation in one X-chromosome in human early embryonic females cells, a transient co-localization of homologous X-chromosomes XICs is required [9]. Further studies showed that before the onset of XCI two homologous X-chromosomes are pairing physically by a specific segment denominated X-pairing region (Xpr) (Fig. 2). This Xpr could potentially play a key role in counting mechanism at the onset of XCI. Xpr is bringing together two XICs and pairing occurs before *XIST* (X inactive specific (non-protein coding) transcript, HGNC:12810) becomes up-regulated on both X-chromosomes; lateron *TSIX* (TSIX transcript, XIST antisense RNA, HGNC:12377) is down-regulated on the future XCI [10, 11]. According to literature the first part of the Xpr aligning involves the *SLC16A2* gene (solute carrier family 16, member 2 (thyroid hormone transporter), HGNC:10923) (Fig. 2) [10]. This association is not disrupted even if a *XIST* heterozygote deletion was present in embryonic stem cells, a finding which means that first steps of XCI (counting and choosing) are independent of *XIST*/*TSIX*/*Xite* region [10]. Besides, murine *Xite* region contains X-inactivation intergenic transcription elements that were shown to regulate the probability of choice [12].

**Fig. 3:**
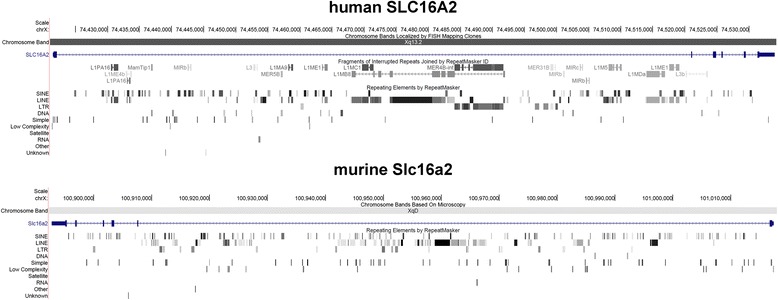
*Slc16a2* gene in humans and mouse. *SLC16A2*/*Slc16a2* gene region is depicted. Note the different transcription directions in mouse and human. Also, human *SLC16A2* contains in its center several “big” LINE elements marked by RepeatMasker

**Fig. 4:**
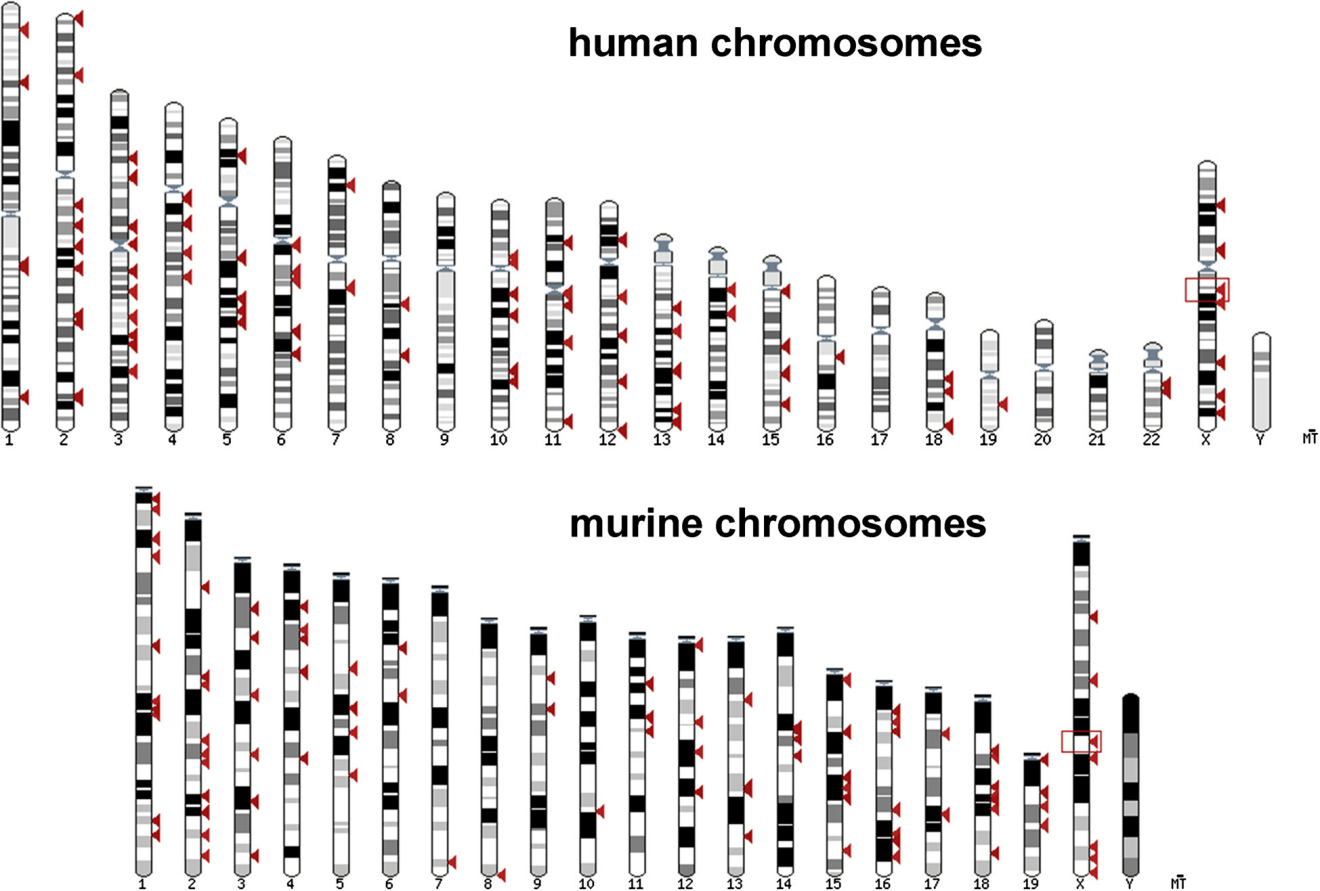
Mapping of the *Slc16a2* gene. Results of Ensebml BLAST search for *SLC16A2*/*Slc16a2* gene sequences in human and mouse. Only 100 hits are presented (including original genomic localization of *SLC16A2*/*Slc16a2* gene). Human *SLC16A2* gene is 40,494 bp long and the size of the regions mapped as homologous goes from 1,161 to 362 bp. These homologous regions map within 43 different genes on 15 chromosomes and to 56 genomic regions on 14 chromosomes. Murine *Slc16a2* is 40,240 bp long and the size of the homologous regions spanned 633 to 532 bp, 30 genes on 14 chromosomes and 70 genomic locations on 19 chromosomes. In human chromosomes 20, 21 and Y did not show any *SLC16A2* homologous sequences

Given all above mentioned findings it can be assumed to be true that X-chromosome pairing and counting are crucial steps for onset of XCI. However, chromosome counting is not only essential for X-chromosomes. Recent studies showed that during early embryogenesis the fetal tissue has features of high chromosomal instability [13–15]. Nonetheless, trisomies and monosomies can be later fixed by these cells [14, 16], indicating for the existence of a counting mechanism for all chromosomes. As gonosomes have autosomal origin, such an autosomal counting mechanism must be the even evolutionary older one. If a single genomic region containing the *SLC16A2* gene can be responsible for sensing and counting of X-chromosomes, we asked ourselves if it there could be *SLC16A2-*like genes/sequences (Fig. 2) on other chromosomes, potentially doing the same job for those autosomes. Furthermore, interactome analysis of *SLC16A2* (evaluation of proteomic interactions of *SLC16A2* using NCBI gene (http://www.ncbi.nlm.nih.gov/gene/6567) and STRING-DB (http://string-db.org) demonstrates the involvement of *SLC16A2* interactome in a variety of processes (i.e. transcriptional regulation), which could be indirectly related to aneuploidy and local epigenetic dysregulations. Closer inside in *SLC16A2*/*Slc16a2* in human and mouse revealed that the human gene contains several “big” LINE elements (long interspersed nuclear elements) (Fig. 3). Accordingly, LINE elements are known to be involved in genome destabilization, which generally result in aneuploidization [17]. An Ensembl BLAST search for human and mouse revealed that similar sequences to *SLC16A2*/*Slc16a2* can be found at different spots in the corresponding genomes (Fig. 4); N.B.: in human we excluded the biggest LINE element from our Ensembl BLAST search. Comparing *SLC16A2*/*TSIX*/*XIST* like sequences (BLAST search) throughout the human genome, homologous regions of them cover all chromosomes equally (Additional files 1 and 2: Figures S1–S2).

## Hypotheses

Taking into account all these findings based on literature and database search, we developed the following hypotheses:

- *SLC16A2* like sequences on autosomes could be and/or encompass the evolutionary older, but functionally active key regions for chromosome counting in early embryogenesis.
- If a single genomic region containing the *SLC16A2* gene is responsible for maintaining “balanced” copy numbers of only one Xa, it is possible that similar sequences or gene/s have the same function for other chromosomes (autosomes); the similarity or homology of these sequences in the genome could be involved in inappropriate chromosomes pairing [18, 19].
- As *SLC16A2*-like sequence could be involved in inappropriate chromosomes pairing, too, they could provide to formation of chromosomal aneuploidies during embryogenesis and cancer development [20, 21].
- “*SLC16A2*/*TSIX*/*XIST* like sequence combinations” are covering the whole human and murine genome, making it plausible that this combination is important for determination of X:autosome ratio in cells and for chromosome counting.

## Discussion and conclusion

Early studies on XCI have been conducted without complete knowledge of human genomic sequences, and as elaborated before, all efforts for finding the “counting region” was focused on a small part of the XIC region. Mechanisms of XCI were extensively studied as early as in 1990. Riggs [20] put forward the idea that along the X-chromosome there are “way station” or “boosters” elements that are facilitating inactivation speeding on the X-chromosome [20]. Furthermore, studies on X:autosome translocation and *Xist* yeast artificial chromosome (YAC) transgenes of the autosome showed that inactivation can spread and silence autosomal genes, too [21, 22]; this was also shown in clinical cases [23]. The inactivation is not as efficiently as inactivation of genes on X-chromosome and it can vary from autosome to autosome. Thus, it was evident that sequence(s) involved in spreading of inactivation is/are not specific to X-chromosomal sequences. Further studies on individual autosomal trisomic female cases showed that XCI is not altered (one Xi and one Xa), while two Xa featured the majority of triploid female embryos [24, 25]. During early years of genetics it was generally assumed that a core of Xi or Barr body was made up from silenced X-chromosomal genes, but 2D and 3D architecture studies revealed higher-level organization of Xi. In general most of the genes (regardless of activity and position on the metaphase chromosome) are arranged in the periphery of the Xi, and most of the noncoding and repetitive sequence reside within the interior of Xi [26].

In summary, facts about X-chromosome counting and XCI are: (i) there is one Xa per diploid set of autosomes in mammalian cells; (ii) before XCI, early in embryogenesis, cells are capable to count chromosomes and to determinate the X:autosome ratio; (iii) on the onset of embryogenesis two (or more) X-chromosomes come in close proximity; (iv) there is a higher-level organization of the Xi (in general noncoding and repetitive sequences inside, while genes are positioned outside). Regarding (iii) and (iv), two opposite “phenomena” were discovered: chromosome territories and chromosome kissing. First one describes how in a nucleus chromosomes are occupying distinct and well-defined territories [27, 28]. The “phenomenon” of two chromosomes coming close together or “chromosome kissing” referrers to inter-chromosomal interactions between pairs of chromosomes or specific parts of them [19].

Chromosome and gene positioning in the nucleus is clearly important for numerous functions. Among others, chromosome counting could be one of the cellular processes that requires specific nucleus architecture in a sense that X-chromosome/s is/are in contact with autosomes.

Sequence similarity across autosomes and X-chromosome(s) could serve as counting mechanism to determine X:autosome ratio, and it could be that some specific genomic regions have the same function for each autosome, too. Consequently, errors during chromosome counting could be the first step in formation of chromosomal aneuploidies during embryogenesis and cancer development. *SLC16A2*/*TSIX*/*XIST* gene like sequence combinations cover the whole genome; thus it may be speculated that they could serve as such check points. Sequence similarities across autosomes and X-chromosome(s) could be prerequisite for pairing and counting mechanisms.

Interestingly, when comparing human and murine X-chromosomes and *SLC16A2*/*Slc16a2* genes localized there, one finds that they have different transcription directions (Fig. 4). If this is meaningful in any way has to be ruled out be further studies. However, it once again raises additional questions about the suitability of mouse as a model for human.

Overall, supportive facts for the here presented hypothesis are that chromosome kissing/counting is important for (i) regulation of gene expression (silencing and activation); (ii) tissue specific transcription; (iii) cell fate and (iv) DNA replication control [19, 29–32]. However, the onset of XCI is most likely not the only example of chromosome kissing. Accordingly, it seems to be necessary to carry out a more generalized search for sequences driving inter-chromosomal interactions. *SLC16A2*/*TSIX*/*XIST* gene like sequence may have to be more in focus of research here.

## Abbreviations

2D and 3D, two and three dimensional; BAC, bacterial artificial chromosomes; BAC FISH, fluorescence in situ hybridization using bacterial artificial chromosomes; BLAST, basic local alignment search tool; DNA, deoxyribonucleic acid; LINE, long interspersed elements; RNA, ribonucleic acid; Xa, active X-chromosome; XCI, X-chromosome inactivation; Xi, inactive X-chromosome; XIC, X inactivation center; Xpr, X-pairing region; YAC, yeast artificial chromosomes

## Additional files

Additional file 1: Figure S1.
*SLC16A2*/*TSIX*/*XIST* like sequences through human genome (chromosomes 1–12). BLAST search result for *SLC16A2*/*TSIX*/*XIST* sequence through human genome showed, that homologous regions of these three genes cover all chromosomes equally. (TIF 2653 kb) Additional file 2: Figure S2.
*SLC16A2*/*TSIX*/*XIST* like sequences through human genome (chromosomes 13–22, X and Y). See figure legend for Additional files 1 and 2: Figures S1-S2. (TIF 3023 kb)
