# Supplementary figures and images for: Thoughts about SLC16A2, TSIX and XIST gene like sites in the human genome and a potential role in cellular chromosome counting
Source: Mol Cytogenet. 2016 Aug 8;9:56. doi: 10.1186/s13039-016-0271-7 (PMC4976476; doi:10.1186/s13039-016-0271-7)

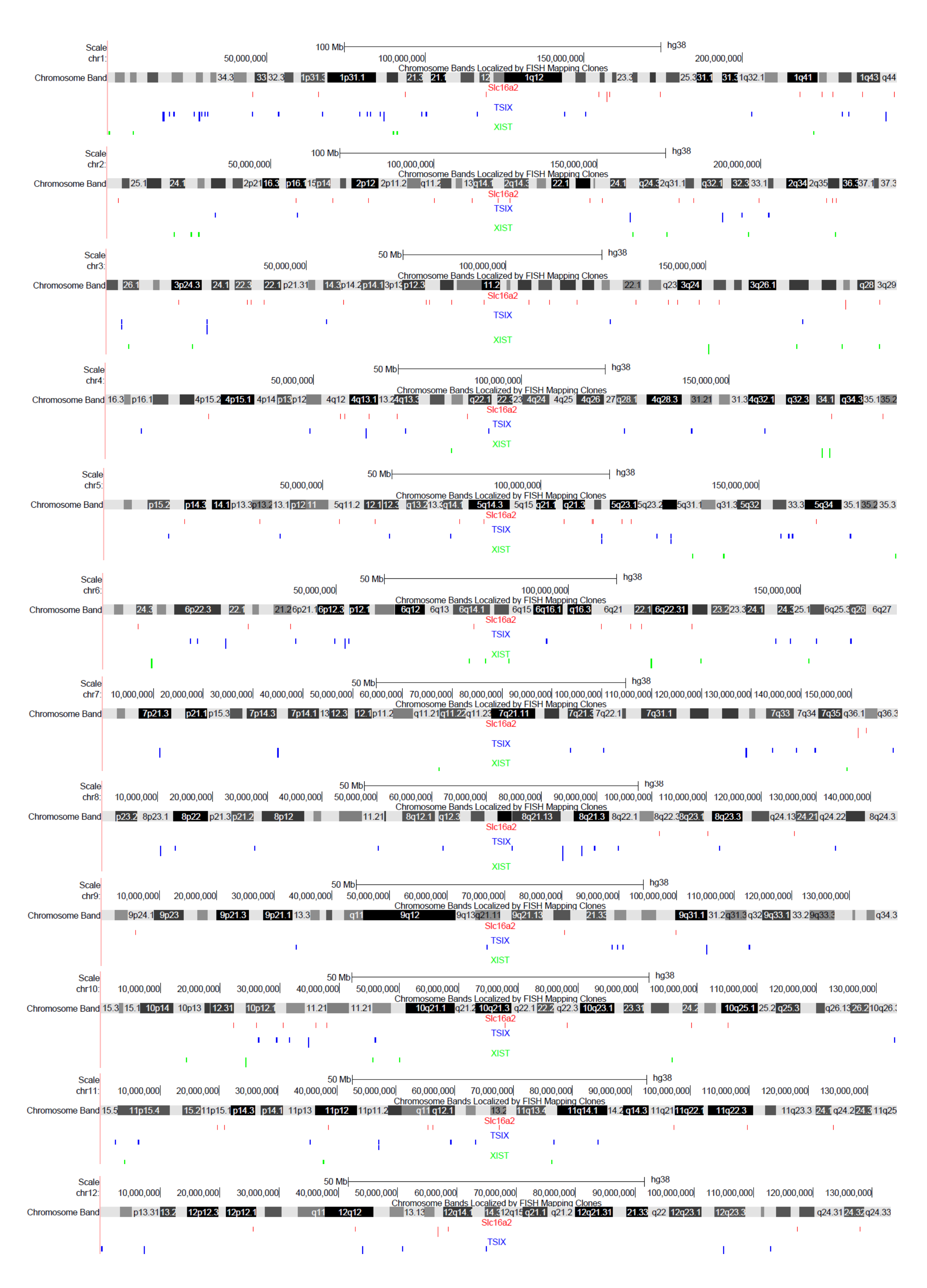

Supplement: Additional file 1: Figure S1. — SLC16A2/TSIX/XIST like sequences through human genome (chromosomes 1–12). BLAST search result for SLC16A2/TSIX/XIST sequence through human genome showed, that homologous regions of these three genes cover all chromosomes equally. (TIF 2653 kb) [file 13039_2016_271_MOESM1_ESM.tif]

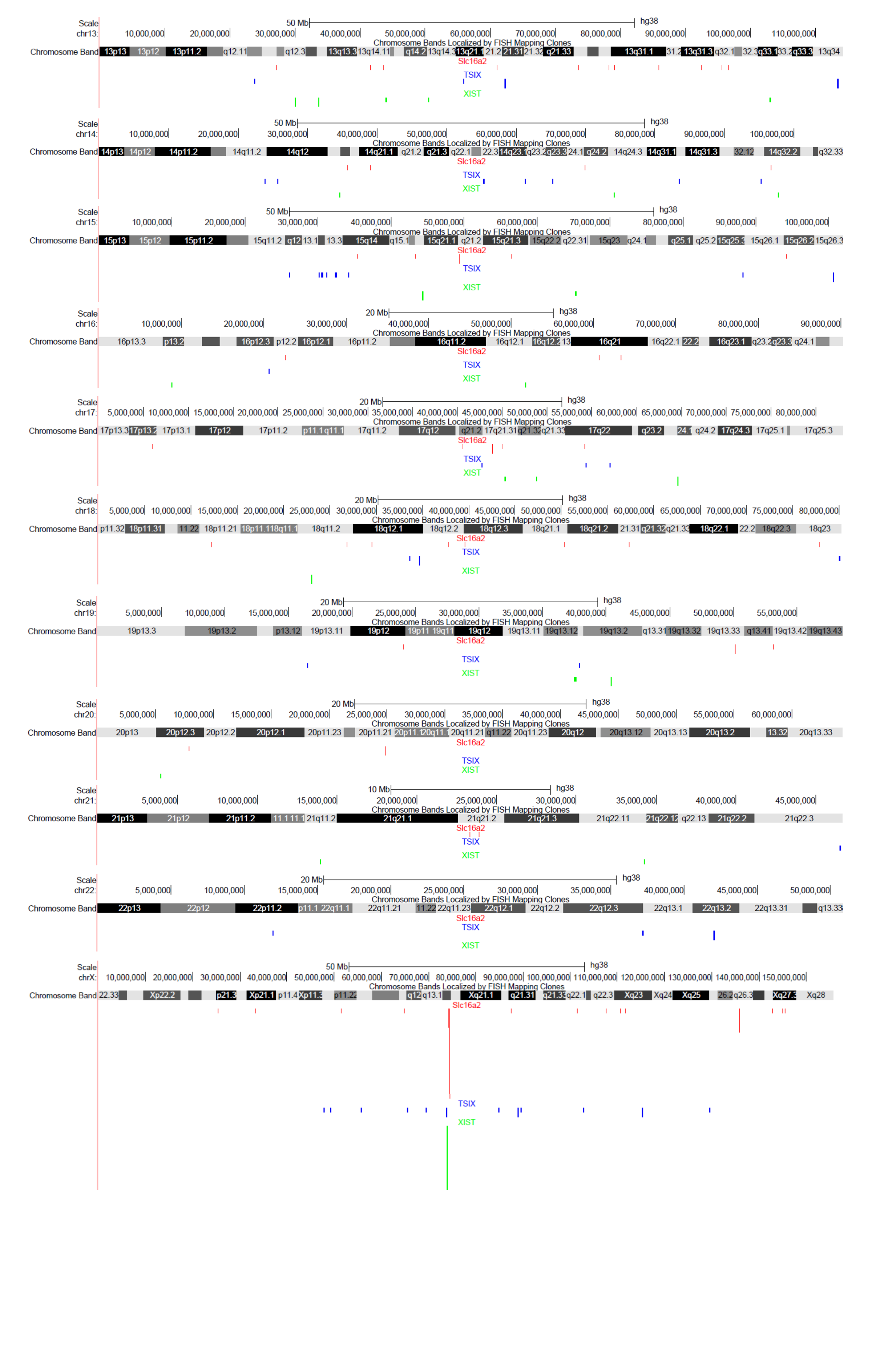

Supplement: Additional file 2: Figure S2. — SLC16A2/TSIX/XIST like sequences through human genome (chromosomes 13–22, X and Y). See figure legend for Additional files 1 and 2: Figures S1-S2. (TIF 3023 kb) [file 13039_2016_271_MOESM2_ESM.tif]
